# Delivery of Monomethyl Auristatin E Using Ionizable Lipid Nanoparticles for B‑Cell Acute Lymphoblastic Leukemia Treatment

**DOI:** 10.1021/acsomega.5c13457

**Published:** 2026-05-25

**Authors:** William H. Pentz, Krystal A. Hughes, Bishal Misra, Srikiran V. Nandigama, Aidan Murray, Aimery Samuelson, Morgan Surface, Rukiye Nur Akpolat-Seker, Boopalan Sivanathan, Sharan Bobbala

**Affiliations:** † Department of Pharmaceutical Sciences, 5631 West Virginia University School of Pharmacy, Morgantown, West Virginia 26506, United States; ‡ School of Medicine, West Virginia University, Morgantown, West Virginia 26505, United States; § Department of Chemistry and Biochemistry, West Virginia Wesleyan College, Buckhannon, West Virginia 26201, United States; ∥ Department of Microbiology, Immunology and Cell Biology, West Virginia University School of Medicine, Morgantown, West Virginia 26506, United States; ⊥ Department of Chemistry, Berea College, Berea, Kentucky 40404, United States; # Department of Clinical Pharmacy, West Virginia University School of Pharmacy, Morgantown, West Virginia 26506, United States; ∇ West Virginia University Cancer Institute, Morgantown, West Virginia 26506, United States

## Abstract

Ionizable lipid nanoparticles (LNPs) are the leading method for clinically delivering nucleic acid cargoes such as messenger or small interfering RNAs. The capacity of LNPs to encapsulate and deliver small molecule therapeutics alongside traditional nucleic acid cargo remains largely unexplored. Beginning the development of a dual delivery approach using LNPs holds significant potential for treating malignancies prone to therapeutic resistance. B-cell acute lymphoblastic leukemia (B-ALL) is a hematologic malignancy with a poor prognosis in patients who experience relapse or are refractory to standard-of-care treatments. Here, we encapsulated the antimitotic agent, monomethyl auristatin E (MMAE), within prefabricated LNPs containing nonfunctional negative control small interfering RNA (siRNA) and evaluated the resulting antileukemic activity in B-ALL preclinical models. We optimized a postfabrication loading technique to encapsulate MMAE within LNPs while maintaining their superior siRNA retention and favorable physicochemical characteristics. Flow cytometry and confocal microscopy studies confirmed that the LNP uptake by B-ALL cells occurred through clathrin-mediated endocytosis and macropinocytosis. MMAE loaded within LNPs demonstrated potent antileukemic efficacy against B-ALL cells *in vitro* compared to the standard-of-care chemotherapeutic vincristine. Furthermore, within a B-ALL human xenograft murine model, MMAE-loaded LNPs exhibited over a 2-fold reduction of leukemic burden in the spleen and the peripheral blood as compared to LNPs without MMAE. Taken together, this study provides the groundwork for developing LNPs as a clinically translatable codelivery platform for chemotherapy and siRNA to improve the treatment of challenging malignancies, such as B-ALL.

## Graphical abstract

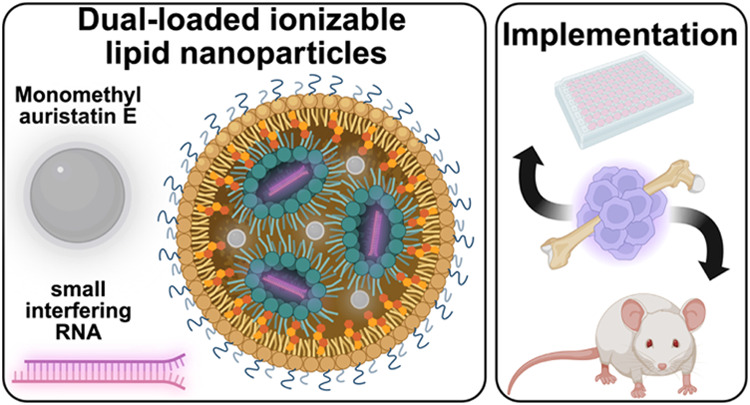

## 1 Introduction

Ionizable lipid nanoparticles (LNPs) are the prevailing nonviral delivery vehicle to clinically achieve safe and efficacious dosing of nucleic acid therapeutics.^1^ To date, the US Food and Drug Administration (FDA) has approved multiple nucleic acid-loaded LNP formulations for prophylactic and therapeutic implementations, including messenger RNA (mRNA) vaccines to protect against respiratory viruses and small interfering RNA (siRNA) therapies for the treatment of hepatic pathologies.^2,3^ Given the clinical success of LNPs for nucleic acid delivery, significant research is underway for their utilization in cancer treatments.^4,5^ Other lipid-based nanoparticles, such as liposomes, already have a long-standing success in clinics, specifically in encapsulating small molecule chemotherapies and enhancing their therapeutic efficacy and tolerability.^6^ For example, liposomal formulations such as Doxil contain a single chemotherapy, doxorubicin, and Vyxeos delivers dual chemotherapies, daunorubicin and cytarabine, to treat multiple cancers.^6,7^ While ionizable LNPs are well characterized for the delivery of nucleic acid therapies, their ability to codeliver chemotherapies remains largely understudied. Simultaneous delivery of siRNA and small molecule therapeutics may hold significant potential to overcome treatment resistance commonly observed in aggressive malignancies.^8^

B-cell acute lymphoblastic leukemia (B-ALL), the main subtype of the most prevalent childhood cancer, exemplifies a malignancy that is known to develop resistance to standard-of-care treatments.^9,10^ Multiagent chemotherapy regimens are used during the primary occurrence of B-ALL and have significantly improved the 5-year overall survival in the pediatric population to close to 90%.^11^ However, leukemic cells that are not eradicated through primary treatment have been identified to reside in unique niches, such as the bone marrow microenvironment, that foster survival and further develop into more aggressive clones displaying chemoresistance.^12–14^ In the 10–20% of the patient population that experience B-ALL relapse or are refractory to initial treatment (R/R), high-dose chemotherapy regimens are frequently used for salvage therapy, requiring constant monitoring due to low tolerability and significant toxicities.^15–19^ While the liposomal formulation of vincristine (Marqibo) demonstrated predictable toxicity and comparable efficacy to high-dose vincristine in R/R ALL patients, it was voluntarily withdrawn from the market due to challenges in patient recruitment for postmarketing confirmatory trials.^20–22^ The loss of Marqibo as a chemotherapeutic option leaves a critical gap in the treatment regimens for ALL before employing complex immunotherapies.

Monomethyl Auristatin E (MMAE) is a small molecule sharing a similar mechanism of action as the standard-of-care antileukemic agent vincristine, both acting by inhibiting the polymerization of microtubules within cells.^23^ Due to its potency, MMAE is currently utilized as a therapeutic payload for antibody-drug conjugates (ADCs) in the clinical treatment of both hematologic and solid tumors.^24^ Improving the development of ADCs capable of achieving high drug-to-antibody ratios without MMAE release or aggregation remains a significant challenge.^25^ The hydrophobicity and ionization transition state of MMAE make it a suitable candidate for loading into lipid-based nanoparticles.^26–28^ Consequently, ionizable LNP platforms hold significant potential to concurrently address two unmet therapeutic needs for B-ALL treatment: delivering a potent small molecule chemotherapeutic while also targeting mechanisms of chemoresistance using a nucleic acid therapy.

In this study, we investigated the capability of ionizable LNPs to effectively load and deliver MMAE in preclinical B-ALL models while preserving the entrapment and retention of a model negative-control siRNA. The cellular uptake and internalization mechanism of our LNP platform was evaluated within primary B-ALL cell lines considered as low-risk (REH cells) and high-risk (SUPB15 cells) for the development of R/R disease based on their molecular subtypes.^10,29,30^ Next, we studied the antileukemic effect of MMAE within LNPs against B-ALL cells *in vitro* compared to vincristine. Finally, we explored the chemotherapeutic effect of loaded-MMAE in the LNP platform within a REH B-ALL human xenograft model. Together, this study presents a novel utilization of ionizable LNPs with high translational potential for B-ALL combination therapy, feasibly containing both chemotherapy and nucleic acid payloads.

## 2 Results and Discussion

### 2.1 MMAE Can Be Readily Loaded within Prefabricated LNPs Containing siRNA

The codelivery of a potent chemotherapeutic, such as MMAE, in tandem to a nucleic acid therapeutic within a single nanoparticle platform necessitates strict control of dosing such that proper efficacy of both treatments is achieved. We have previously shown that rapid fabrication of ionizable LNPs can be achieved using flash nanoprecipitation (FNP) with a confined impingement jet mixer, capable of efficiently encapsulating mRNA and siRNA therapies, alongside small molecule payloads with favorable physicochemical characteristics.^31^ Initial attempts to load MMAE into ionizable LNPs were performed by adding the chemotherapy directly during the self-assembly process. Negative-control siRNA (NC-siRNA) was dissolved in sodium citrate buffer (pH 4) and rapidly mixed with the organic phase containing lipid components and MMAE. It is important to note that nucleic acid payload allows morphological stability of LNPs, and therefore, we chose to employ NC-siRNA in all our formulations while encapsulating MMAE. MMAE concentrations were quantified using liquid chromatography-tandem mass spectrometry (LC-MS/MS) before and after removal of unloaded MMAE using centrifugal filtration, leading to an overall MMAE encapsulation efficiency of 3.3% with loading during the FNP process (Figure S1A). We suspected this low encapsulation could be attributed to the ionization state (p*K*_a_) of MMAE, which is predominantly ionized in acidic conditions, reducing any favorable hydrophobic interaction between MMAE and the LNPs. It is important to note that self-assembly of LNPs necessitates acidic formulation conditions to enable electrostatic interaction between ionizable lipids and the nucleic acids.^32^ To ensure the favorable loading of nucleic acids was maintained, we altered our focus to enhancing MMAE loading postfabrication of the siRNA-LNPs in a more basic environment, utilizing the hydrophobic nature of MMAE to partition the chemotherapy into the hydrophobic spaces of the LNP.

Based on murine studies with antibody-drug-conjugates, the maximum tolerable dose (MTD) of unbound MMAE is reported with doses around 0.5–1.0 mg/kg or 0.2–0.5 mg/kg for single and multiple intravenous administration, respectively.^33–35^ Since siRNA therapeutic regimens primarily follow multiple doses, we opted for low drug-to-lipid (D/L) ratios ranging from 0.003 to 0.06, positioning the finalized formulations below the multidose MTD of MMAE while maintaining achievable therapeutic dosing with siRNA in future iterations of the platform. Postfabrication loading at D/L ratios between 0.003 and 0.06 with prefabricated siRNA-LNPs buffered against phosphate-buffered saline (PBS; 7.4) resulted in low MMAE encapsulation efficiency, ranging from 2.2 to 3.3% (Figure S1B). While there was no observed trend between the D/L ratio and encapsulation efficiency, a strong linear correlation (*R* ^2^ = 0.99) was present between the D/L ratio and drug-loading capacity (DLC) for the LNP platform. Even at the highest D/L ratio attempted of 0.06, the DLC achieved was 0.2%.

To improve our postfabrication loading method, we suspected that the ionization of MMAE was significantly impacting loading. The structure of MMAE has a secondary amine in the α-position to an adjacent amide, which will primarily be ionized at physiologic pH. Importantly, the electron-withdrawing effects of this nearby carbonyl group reduce the p*K*_a_ of the amine, which typically ranges for secondary amines around a pH of 9 to 11. Therefore, fabricated siRNA-LNPs were buffered against a more basic 0.1 M bicarbonate-carbonate buffer (pH 10.6–11.0) rather than PBS. At a pH at least 1 log unit above the p*K*_a_, it is expected that over 90% of MMAE would exist in its un-ionized state, which is critical for membrane permeability.^36^ In a direct comparison study at a D/L ratio of 0.015, high-pH buffered siRNA-LNPs increased MMAE encapsulation efficiency by a factor of 3 and increased DLC by a factor of 2.9 compared to PBS-buffered LNPs, corroborating our hypothesis (Figure S1A).

To mitigate loss and improve encapsulation efficiency of MMAE within our siRNA-LNPs, we speculated that increasing the amount of lipid present during postfabrication loading plays an important role in providing a thermodynamic gradient to suitably drive MMAE within the LNPs. MMAE is highly hydrophobic, with a computed log *P* of 4.1 (XLogP3 3.0), making the hydrophobic interior of the LNP a more entropically favorable environment compared to the surrounding aqueous buffer.^37^ Notably, we observed a strong positive correlation between the total lipid mass present during loading and the MMAE encapsulation efficiency, a trend that remained regardless of the D/L ratio (Figure S1C). This finding not only demonstrates the importance of creating a sufficient thermodynamic environment for loading MMAE, it also presents the feasibility of further scaling this fabrication process with enhanced loading efficiency. By combining these optimizations, our final protocol utilized bicarbonate-carbonate dialyzed at a D/L ratio of 0.06 with a mass of 10–15 mg total lipid during loading (Figure 1A). These conditions achieved coloaded MMAE-siRNA LNPs (MMAE-LNPs) with an encapsulation efficiency of 27.3 ± 1.5% (Figure 1B).

**Figure 1:**
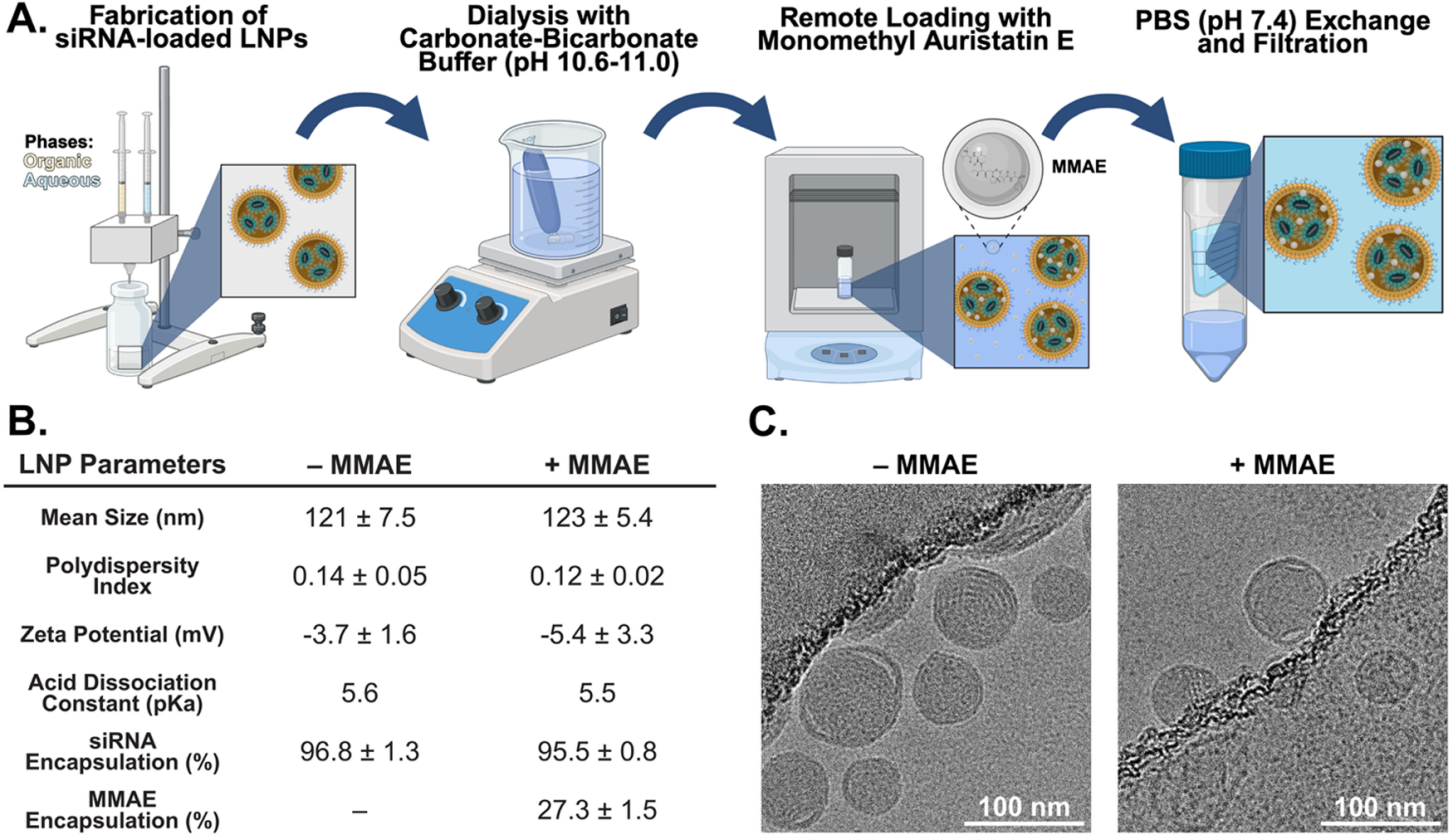
Approach to loading MMAE in siRNA-LNPs and their physicochemical characterization. (A) Schematic of optimized postfabrication loading approach of MMAE into LNPs. (B) LNP characterization represented without (−MMAE) and with (+MMAE) MMAE loading. Mean hydrodynamic diameter and polydispersity of LNPs were determined using dynamic light scattering. The zeta potential of LNPs was determined using electrophoretic light scattering and represented in millivolts (mV). The apparent ionization state (p*K*_a_) of siRNA-LNPs ± MMAE was extrapolated from the normalized fluorescence of 50% using a TNS (6-p-Toluidino-2-naphthalenesulfonic acid) fluorescent assay. The percentage of relative siRNA encapsulated after LNP fabrication was determined using the Quantifluor RNA system-based assay. The percentage of MMAE encapsulated was quantified utilizing LC-MS/MS, with encapsulation representing the amount of MMAE in LNPs after filtration versus the amount that was initially present during loading. All data represented as mean ± SD (*n* = 3). (C) Morphology of LNPs was visualized using Cryo-transmission electron microscopy. The scale bar is 100 nm. Panel A was created using BioRender (https://BioRender.com/3bpo427).

With the same ionizable LNP composition used clinically for siRNA delivery, our optimized loading method for MMAE achieved a DLC of 1.5 ± 0.1%, 7.5-fold higher than our initial findings.^38^ To the best of our knowledge, the loading of MMAE in addition to nucleic acid therapeutics has not been reported for ionizable LNPs. The loading of unmodified MMAE in liposomes and polylipopeptide micelles has been previously reported with a DLC ranging from 0.1 to 5%, aligning well with our current findings.^39,40^ The amount of loaded MMAE within our platform is well-suited for evaluating standard preclinical siRNA dosing regimens.^8,41^ While further enhancement of the MMAE DLC within the platform may be required for clinical translation, it must be contextualized with the therapeutic windows for both MMAE and the utilized functional siRNA. Treatment regimens with siRNA generally require multiple doses to provide transient knockdown of a desired gene target. Consequently, a low coloaded dose of MMAE would be favorable during future studies evaluating possible synergies with functional siRNA while minimizing any adverse events associated with MMAE toxicity.

### 2.2 MMAE-Loading Does Not Impact Physicochemical Characteristics or siRNA Loading in LNPs

Loading large amounts of compounds within lipid-based nanoparticles, such as liposomes and nanostructured lipid carriers, has been previously reported to alter the morphology, stability, and overall size of nanoparticles.^42,43^ After thorough characterization, MMAE-LNPs exhibited comparable physicochemical characteristics to siRNA-LNPs (Figure 1B). Dynamic light scattering (DLS) revealed that LNPs were monodisperse, which was demonstrated by a polydispersity index of less than 0.3, and showed a hydrodynamic diameter between 120 and 130 nm. Cryo-transmission and transmission electron microscopy images corroborated our findings with DLS and confirmed that LNPs retained spherical morphology after MMAE loading (Figures 1C and S2B).

The endosomal escape of siRNA is influenced by cationic headgroups on LNPs interacting with the lipid membrane of late endosomes and lysosomes in their physiologic acidic conditions.^44^ While siRNA release into the cytoplasm of cells is a desirable and intended outcome of LNPs, positively charged headgroups on LNPs while in systemic circulation can readily interact with the negatively charged membranes of off-target cells, resulting in unwanted, cytotoxic effects.^45,46^ The utilization of an ionizable lipid with a zwitterion headgroup, such as D-Lin-MC3-DMA (MC3) used in this study, provides a favorable medium that mitigates undesirable cytotoxicity in circulation while inducing endosomal escape of nucleic acid cargo after endocytosis. Accordingly, the effects of loading MMAE within siRNA-LNPs on the platform’s apparent p*K*_a_ and surface charge at physiological pH were evaluated. A 6-p-Toluidino-2-naphthalenesulfonic acid assay was performed on siRNA-LNPs without and with MMAE, which showed apparent p*K*_a_ values of 5.6 and 5.5, respectively. These findings are in agreement with apparent p*K*_a_ values of other MC3-based LNPs with deviations from varying formulation compositions, concentration utilized during testing, and different manufacturers.^31,47^ Electrophoretic light scattering showed that siRNA-LNPs produced with or without MMAE were neutrally charged with a zeta potential ranging between 0 and −10 mV at physiologic pH^48^ (Figure 1B).

Next, we evaluated the relative siRNA encapsulation efficiency in LNPs before and after the loading process of MMAE. All fabricated LNPs showed a relative siRNA encapsulation efficiency greater than 95%, and following the MMAE loading process and dialysis, 92% of the encapsulated siRNA was retained in LNPs (Figures 1B and S2A). Additionally, shelf life stability and the release of MMAE from LNPs were considered. Prolonged retention of siRNA content within LNPs has been previously reported; however, reports on the retention of additional cargo in RNA-loaded LNPs are minimal.^49^ MMAE-LNPs were characterized immediately after creation and then after 1 month stored at 4 °C. MMAE-LNPs maintained 95% of their original size and contained 91% and 62% of their original siRNA and MMAE content, respectively (Figure S3A,B). To assess the platform’s stability in simulated physiologic conditions, kinetic release studies were performed and demonstrated that less than 15% of the encapsulated MMAE payload was released from MMAE-LNPs over a 48-h incubation within fetal bovine serum at 37 °C (Figure S3C). This observation may be attributed to MMAE tightly packing inside hydrophobic spaces of the LNPs, which is in line with recent studies showing minimal release of the small molecule payload, dexamethasone, from mRNA-loaded LNPs in similar physiologic conditions.^50^ While these findings highlight the minimal change in physicochemical characteristics and stability of our MMAE-LNP platform for immediate use, future iterations of the platform should consider additional techniques to prolong the retention of MMAE during shelf life stability. This includes but is not limited to (i) lyophilization of LNPs using a cryoprotectant immediately after the preparation, (ii) storage of LNPs in the presence of cryoprotectant in −20 or −80 °C, or (iii) concentrating LNP suspensions to lower diffusion of encapsulated cargoes into the aqueous vehicle.

### 2.3 siRNA-LNPs Display *In Vitro* Uptake by B-ALL Cells via Clathrin-Mediated Endocytosis and Macropinocytosis

LNP uptake is facilitated by many cell types, including immune cells, through clathrin-mediated endocytosis; however, there is limited research validating this mechanism in B-ALL cell lines.^51^ Additional mechanisms of uptake, such as macropinocytosis have been shown to play a critical role in LNP uptake and are known to be performed by B-cells with varying reports of capacity.^52–54^ We first evaluated the inherent toxicity of our LNP platform loaded with NC-siRNA in B-ALL cells. After 72 h incubation, the viability of REH and SUPB15 cells remained above 80% compared to our PBS control with loaded NC-siRNA concentrations ranging from 1 to 100 nM in the well, and demonstrates the siRNA-LNP platform exhibits minimal toxicity without MMAE loading (Figure S4). These amounts were chosen as they represent a conventional range of siRNA concentrations that are widely used for functional transfection or gene knockdown studies.^8,41^

To provide further insight into possible B-ALL LNP uptake mechanisms, siRNA-LNPs were coloaded with the hydrophobic dye, DiD. We pretreated B-ALL cells with 25 or 50 μM chlorpromazine (CPZ), an inhibitor of clathrin-mediated endocytosis,^55^ or 50 μM amiloride (AMIL), an inhibitor of macropinocytosis,^56^ followed by incubation with LNPs. Flow cytometry analysis revealed that LNP uptake after 24 h in inhibitor-treated REH cells was significantly reduced. As compared to PBS control-treated cells, a significant decrease in the measured DiD median fluorescent intensity (MFI) of 41% and 37% by AMIL and CPZ inhibition, respectively (Figure 2A). Similarly, in SUPB15 cells, a significant reduction in MFI of 35% was seen with AMIL incubation and a 47% reduction with CPZ incubation compared to the PBS-treated cells (Figure 2C). These findings were further confirmed with confocal microscopy, which showed a reduction in intracellular localization and fluorescent intensity of LNPs after treatment with uptake inhibitors in both REH and SUPB15 cells (Figure 2B,D). It has been reported that amiloride and compounds with similar modes of action can inhibit the dynamin-dependent pathway known as fast endophilin-mediated endocytosis (FEME). FEME activity is nonconstitutive in B-cells, with activation occurring after engagement with antigen-binding domains, most notably the B-cell receptor.^57,58^ Because the LNPs used in this study lack the antigens to activate FEME, the capacity of amiloride to inhibit FEME is less significant compared to its ability to inhibit macropinocytosis. However, this highlights the importance of recognizing the specificity of the small molecules being utilized for *in vitro* nanoparticle uptake studies, and reinforces the need for developing methods to evaluate nanoparticle uptake mechanisms *in vivo*.^55^

**Figure 2:**
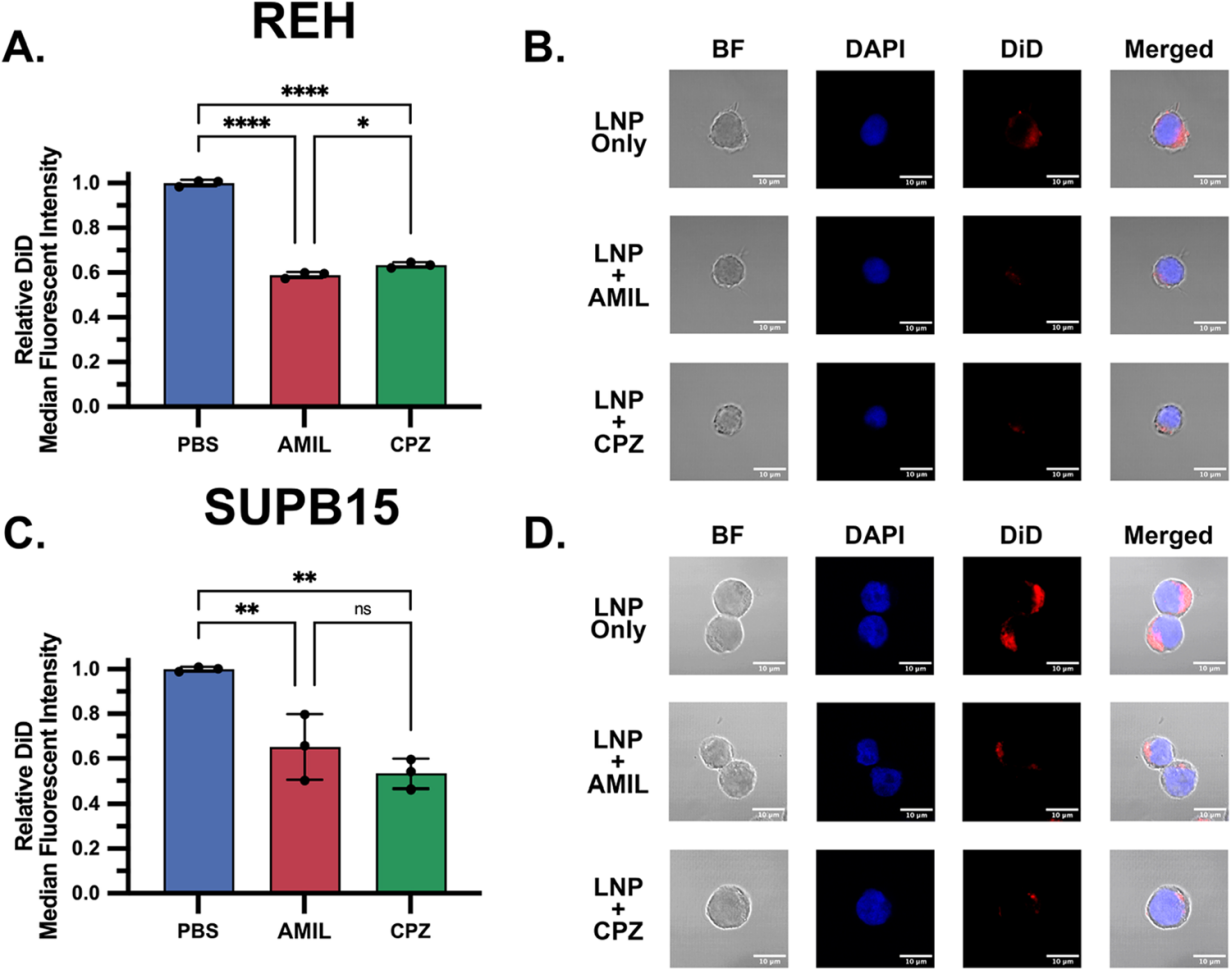
Inherent uptake of DiD-loaded LNPs in REH and SUPB15 cells. DiD-loaded LNPs with negative-control siRNA were incubated in wells containing REH (A−B) or SUPB15 (**C–D**) cells for 24 h following 30 min incubation with PBS, amiloride (AMIL), or chlorpromazine (CPZ). (A and C) Uptake of LNPs was measured by flow cytometry and indicated by the relative median fluorescent intensity of DiD between preincubation conditions. Data represented as mean ± SD (*n* = 3). Significance determined with one-way ANOVA with Tukey’s multiple comparisons test, *p < 0.05, **p < 0.01, and ****p < 0.0001. (B and D) Localization of DiD-loaded LNPs at a 24-h time point visualized utilizing confocal microscopy. DiD intensities are directly comparable only within their figure panels. Scale bars represent 10 μm. Additional images are present in Figure S6.

Intracellular delivery of siRNA is an important prerequisite to the successful knockdown of a gene target of interest. We therefore studied the time-dependent cellular uptake and intracellular localization of Cy5-siRNA loaded within our LNP platform to establish its ability to deliver siRNA payloads within B-ALL cells. Flow cytometry analysis demonstrated a continual increase in MFI over 24 h, with a 4-fold and 2-fold increase in relative MFI between 24 and 4 h time points in REH and SUPB15 cells, respectively (Figure 3A,C). Confocal microscopy at 24- and 4-h time points confirmed intracellular localization of the siRNA payload in both REH and SUPB15 cells (Figure 3B,D). Subcellular localization of Cy5-siRNA in REH cells after 24 h siRNA-LNP incubation was additionally evaluated with imaging flow cytometry. Importantly, 77 ± 14% of the intracellular Cy5 intensity within REH cells was outside of lysosomes by 24 h, providing further insight into the release of siRNA cargo in B-ALL cell lines (Figure S5A,B). Collectively, these findings demonstrate the ability of B-ALL cells to internalize LNPs, which delivered both siRNA and hydrophobic cargo precisely inside the cells. The performed endocytic inhibitor studies support the involvement of both clathrin-mediated endocytosis and macropinocytosis in the cellular uptake of LNPs in B-cell malignancies, underlining the need to evaluate additional endocytic mechanisms in the future.

**Figure 3:**
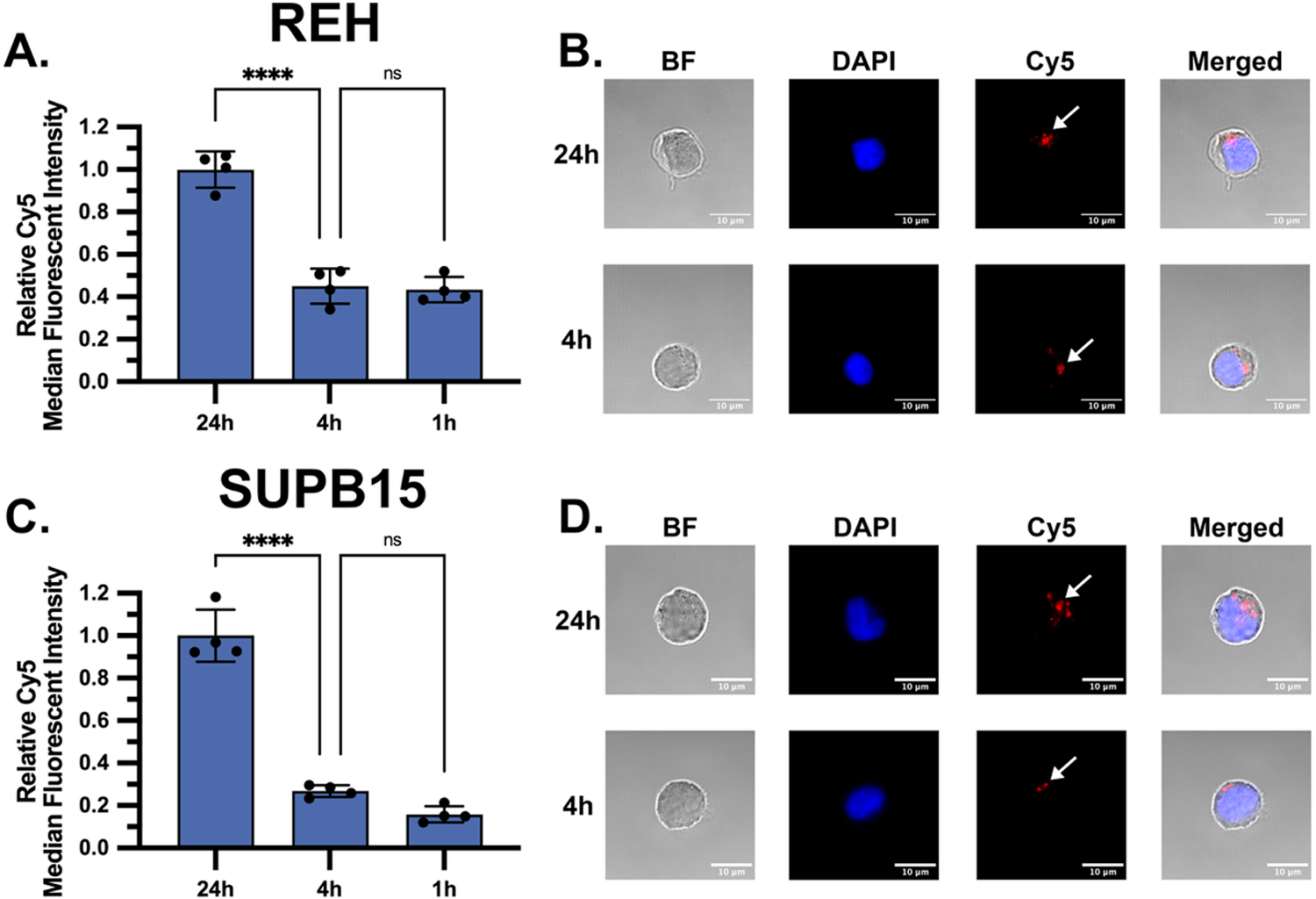
Localization of siRNA after LNP uptake in REH and SUPB15 cells. B-ALL cells were incubated with LNPs containing Cy5-conjugated siRNA at 1, 4, and 24 h time points in REH (A−B) and SUPB15 (**C–D**) cells. (A and C) Cumulative uptake of siRNA was measured flow cytometry and represented as relative Cy5 median fluorescent intensity at respective time points. All data are represented as mean ± SD (*n* = 4). Significance determined with one-way ANOVA with Tukey’s multiple comparisons test, ****p < 0.0001. (B and D) Visualization of intracellular localization of Cy5-siRNA after incubation with LNPs at 24- and 4-h time points with confocal microscopy. White arrows represent definitive areas of intracellular Cy5-siRNA. Cy5 intensities are directly comparable only within their figure panels. Scale bars represent 10 μm. Additional images are present in Figure S7.

### 2.4 MMAE-LNPs Exhibit Potent *In Vitro* Antileukemic Activity against B-ALL Cell Lines

Unconjugated MMAE has well-characterized half-maximal inhibitory concentrations (IC50s) with subnanomolar to nanomolar ranges in numerous B-cell lymphomas.^26^ Vincristine and MMAE both exhibit similar mechanisms of action as antimitotic agents. Given that vincristine is a standard-of-care agent for the treatment of B-ALL, we directly compared the *in vitro* cytotoxicity of MMAE, MMAE-loaded LNPs, and vincristine after 48-h incubation in B-ALL cell lines (Figure 4A,B). In both B-ALL cell lines tested, MMAE or MMAE-LNPs were found to have an IC50 ranging from 1 to 2 nM. Interestingly, vincristine was less potent than MMAE with IC50s of 99.5 nM and 79.1 nM in REH and SUPB15 cells, respectively (Figure 4C). Our findings further corroborate the significant potency of MMAE in B-cell malignancies and are consistent with reported B-ALL toxicities for vincristine, with IC50 values ranging from 30 to 300 nM.^29,59–62^

**Figure 4:**
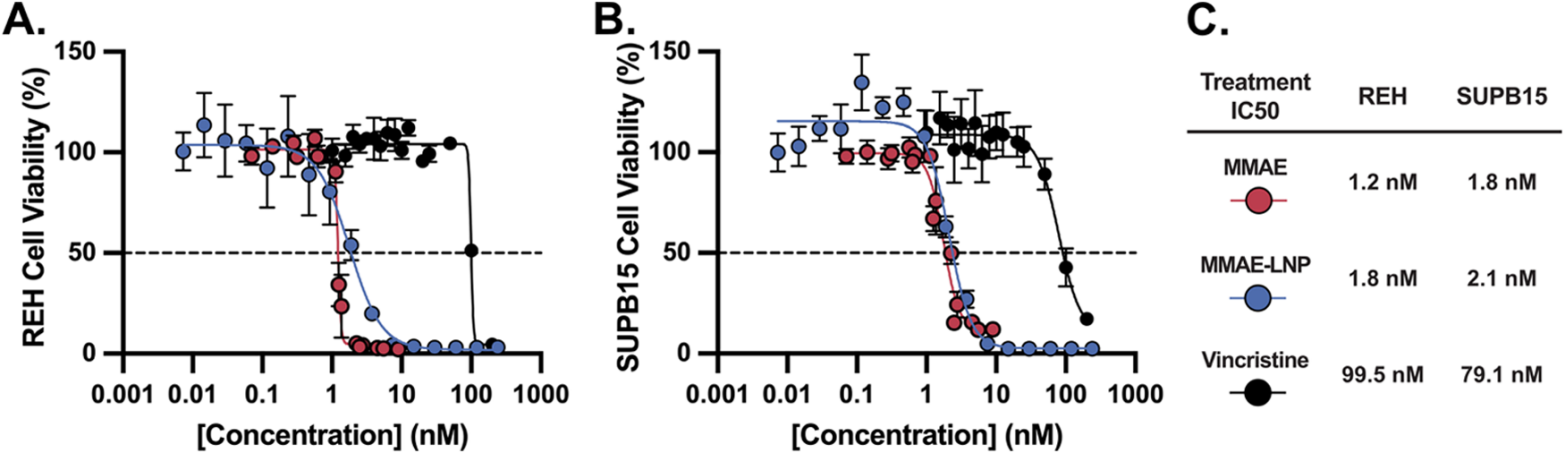
*In vitro* cytotoxicity assays of unloaded MMAE (red), MMAE-loaded LNPs (blue), and unloaded vincristine (black) after 48 h incubation with (A) REH and (B) SUPB15 cells, respectively. Viability was determined using CellTiter-Glo 2.0 and normalized against PBS-treated controls. Data represented as mean ± SD (*n* = 3–4). (C) Table shows the half-maximal inhibitory concentration (IC50) for each treatment in nM. A sigmoidal, nonlinear regression was used to extrapolate IC50s.

To evaluate if the *in vitro* nanomolar potency of MMAE was preserved when loaded within our LNP platform, an extra-sum-of-squares F-test was performed for MMAE and MMAE-LNP curves. While the IC50 values for MMAE and MMAE-LNPs were statistically distinct in both REH and SUPB15 cells (*p* < 0.05), the difference in potency is less than 5 nM, indicating only a minor alteration in chemotherapeutic potency after LNP loading that is biologically negligible. From our kinetic release studies, less than 15% of MMAE is released in serum-like conditions over a 48 h period (Figure S3C). This is consistent with our nanoparticle uptake studies, which suggest MMAE is likely released intracellularly to maintain its high potency. As proposed for siRNA release by endosomal escape, ionization of the LNP platform during endosomal acidification destabilizes the core LNP structure, resulting in the release of both siRNA and MMAE cargo. Electrostatic interactions between the protonated ionizable lipids and the anionic endosomal bilayer subsequently allow for the localization of LNP cargo into the cytoplasm, facilitating the cellular action of MMAE.^63^ In the future, rapidly developing techniques such as imaging flow cytometry should be utilized to more precisely contextualize the intracellular corelease of siRNA and MMAE spatiotemporally.

### 2.5 MMAE Encapsulation Does Not Alter the Biodistribution of LNPs *In Vivo*

Any altered surface properties of nanoparticles following drug encapsulation may impact their inherent biodistribution.^64,65^ To understand this, we evaluated the potential impact of MMAE loading on LNP biodistribution in 6-week-old BALB/c mice. The near-infrared dye tracer, indocyanine green (ICG), was incorporated into LNPs to track nanoparticle biodistribution through IVIS imaging. Mice were intravenously injected with a single dose of ICG-LNPs with or without MMAE-loading. Dosing was matched on the content of NC-siRNA present (2.0 mg/kg) in both formulations, with MMAE-loaded LNPs receiving 0.3 mg/kg of MMAE. Following 4 and 24 h after administration, animals were euthanized, perfused, and the brain, heart, lungs, liver, spleen, kidneys, tibia, and femur were harvested to evaluate the ICG fluorescence with IVIS imaging.

Background-corrected radiant efficiency from the visceral organs and the brain indicated no significant difference in the distribution of organ fluorescence 4 h after administration of ICG-LNPs, regardless of MMAE loading, with predominant accumulation observed in the liver followed by the spleen, respectively (Figures S8–9). The adsorption of serum proteins, such as apolipoprotein E, is believed to be a significant cause for liver tropism and accumulation seen with many nanoparticles.^66^ The high level of tissue perfusion in the liver and spleen may also contribute to MMAE-LNP accumulation in addition to the physiologic role of these organs as primary sites for the mononuclear phagocyte system (MPS).^32,66^ Murine pharmacokinetic studies have additionally found the liver as the primary site for MMAE metabolism and elimination when unbound or conjugated to ADCs, indicating the liver as an inherent site of compound accumulation in addition to other perfused tissues.^67,68^ Overall, localization of MMAE-LNPs in the liver and spleen could be advantageous to the treatment of B-ALL because these extramedullary sites are importantly known to be reservoirs for residual B-ALL malignancy.^69^ As extrahepatic targeting and cellular specificity remain a significant challenge for LNP platforms, the observed findings hold promise for developing treatments against hematologic malignancies, like B-ALL, without immediate implementation of further specialization, such as active-targeting approaches.^70,71^

To evaluate the duration of organ accumulation and distribution, we compared the radiant efficiencies of ICG-LNPs loaded with MMAE at 4 and 24 h after a single intravenous injection (Figure 5A). The ICG signal was apparent through fluorescence measured in soft tissue organs and bone marrow with the signal being brighter at 4 h as compared to 24 h following administration, indicating clearance of LNPs with time (Figure 5B–C). Likewise, the quantified total radiant efficiencies were significantly higher at 4 h compared to 24 h in all organs (Figure 5D). These findings are corroborated by a study where radiolabeled lipids were utilized to evaluate LNP biodistribution and pharmacokinetics, identifying that LNPs comprised of the same lipid components had a half-life of 3 to 5 h after single intravenous administration in mice.^72^ While at the cost of potentially altering transfection capabilities, utilizing pegylated-lipids with longer alkyl-chains has been shown to prolong the circulation time of LNPs, enhancing the ability to target extrahepatic tissues.^6,73,74^ MMAE-LNPs further warrant studies optimizing circulation times and specificity for primary or secondary sites of B-ALL malignancy while also considering the transfection efficiency of siRNA.

**Figure 5:**
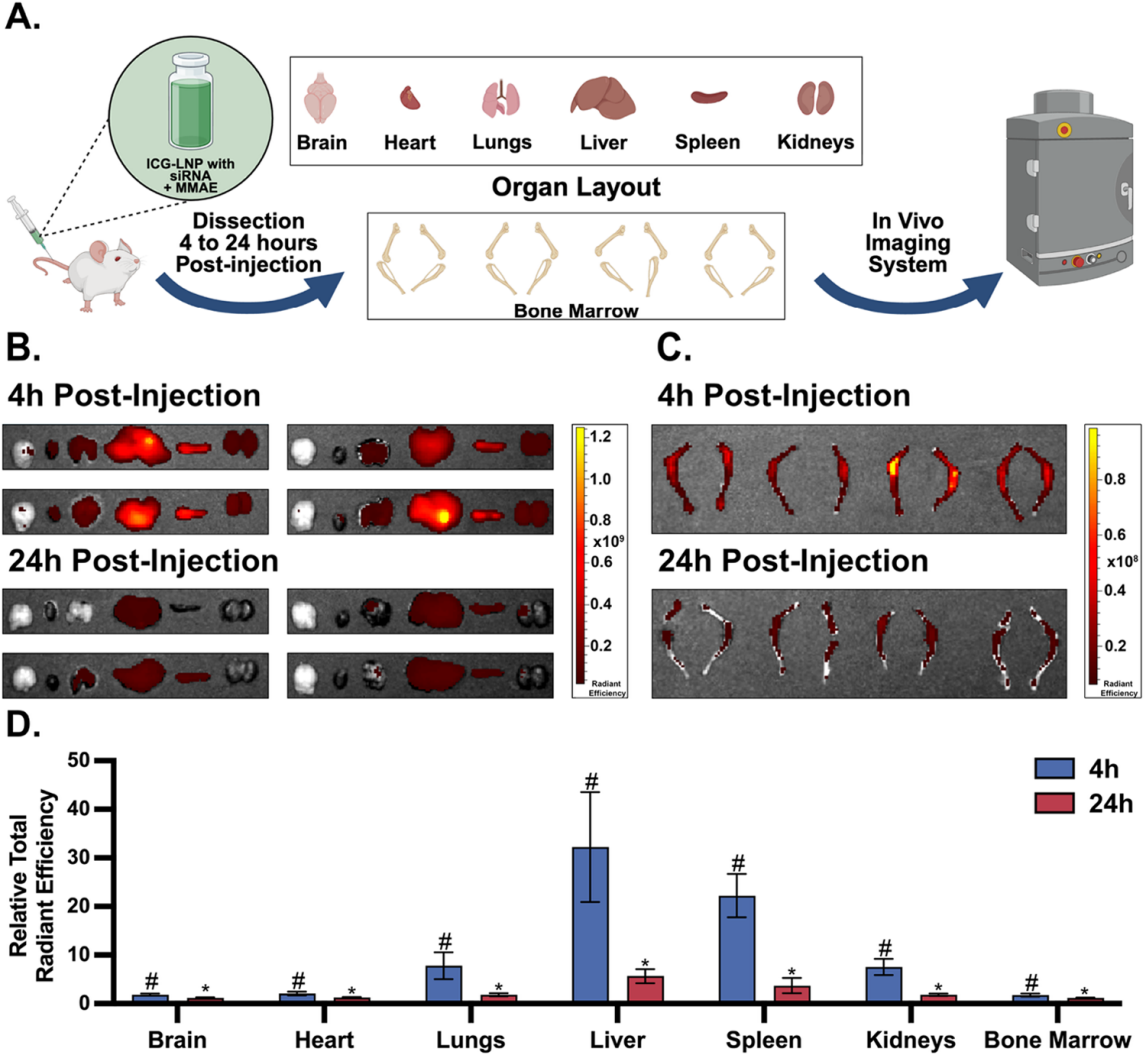
(A) Time-dependent in vivo biodistribution of MMAE-LNPs in BALB/c mice. Mice were intravenously injected with ICG-labeled LNPs containing both MMAE (0.3 mg/kg) and negative control siRNA (2.0 mg/kg) where *ex vivo* analysis was performed at 4 h (*n* = 4) and 24 h (*n* = 4) time points. Visualization of radiant efficiency ([p/sec/cm^2^/sr]/[μW/cm^2^]) for (B) brain and visceral organs along with (C) bone marrow. (D) Quantitative representation of organs was determined by normalizing the total radiant efficiency to the averaged PBS control group (*n* = 3) for each organ. Significance within organ groups was determined with one-way ANOVA and posthoc Tukey’s multiple comparison (#*p* < 0.01 vs PBS, *p < 0.01 vs 4h). All data are represented as mean ± SD (*n* = 4). Panel A was created using BioRender (https://BioRender.com/hw8bm27).

### 2.6 MMAE-LNPs Exhibit a Dose-Dependent Reduction in Leukemic Burden in Human Xenograft B-ALL Mice

To evaluate the translative potential of MMAE in treating B-ALL *in vivo*, we utilized 6-week-old, NSG mice engrafted with human REH B-ALL cells. Xenograft engraftment rates in NSG mice with REH cells have been shown to be high and can be readily monitored through clinical signs such as hind limb paralysis and weight loss.^75–77^ Given the narrow therapeutic index of MMAE, we performed a preliminary study to evaluate the inherent toxicity of free MMAE in our B-ALL human xenograft model. Multidose regimens with MMAE have been reported to show signs of systemic toxicity above MMAE doses of 0.2 mg/kg.^34,35^ We therefore chose to intravenously administer free MMAE at 0.1 mg/kg, given every 3 days for a total of four doses beginning one-week postengraftment. Weights were carefully monitored, and by the end of the treatment period, mice treated with MMAE experienced an overall decrease of 10% their original body weight, significantly deviating from the untreated group’s mean weight (Figure S10). This abrupt weight loss can be attributed to systemic toxicity that is frequently reported with MMAE in addition to signs of peripheral neuropathy and pancytopenia.^33–35^

Multidose regimens of MMAE-LNPs were administered at lower MMAE doses of 0.02 and 0.05 mg/kg. This conservative dosing approach was utilized to mitigate the chance of hepatotoxicity being a limiting effect, given the predominant LNP accumulation in the liver as observed in our biodistribution studies. For our control group, mice were given only NC-siRNA-loaded LNPs with 0.0 mg/kg MMAE (Figure 6A). All formulations were intravenously administered and contained a dose of NC-siRNA ∼0.3 mg/kg. Following 24 days postengraftment, the tumor burden within the bone marrow, along with the additional leukemic sites, such as the spleen and blood, was evaluated for all groups with flow cytometry.

**Figure 6:**
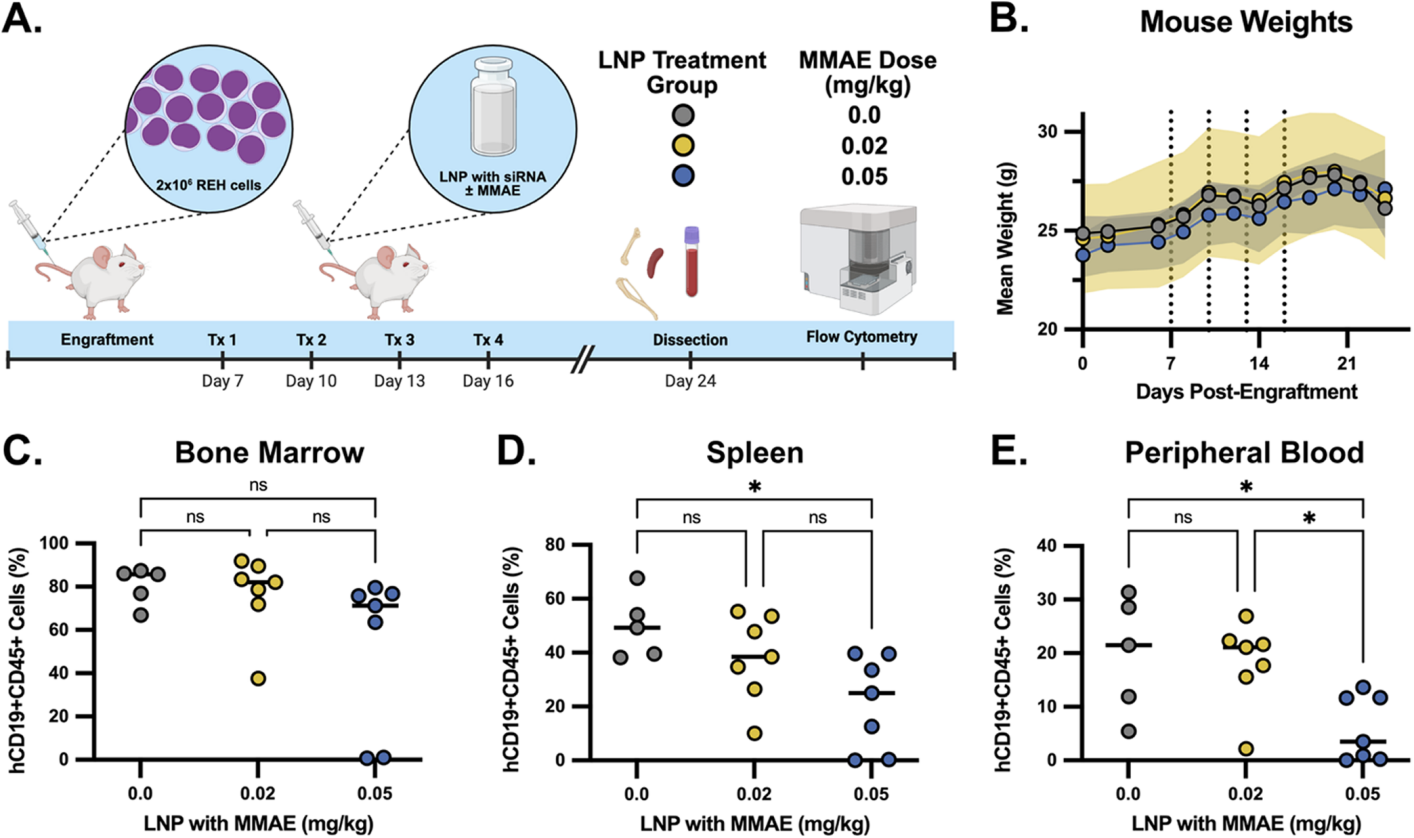
Xenograft leukemic burden study in NSG mice. 6-week-old male mice were intravenously injected with 2 million REH cells. (A) Treatments were administered intravenously via tail vein starting 7 days postengraftment and continuing for 3 additional injections on days 10, 13, and 16. LNP with negative control siRNA containing 0.0 mg/kg MMAE was used as a control treatment (*n* = 5). Other treatments included LNPs with 0.02 mg/kg (*n* = 7) and 0.05 mg/kg (*n* = 7). All mice were euthanized and dissected once the clinical signs in the control group were indicated such as hind limb paralysis or weight loss of 10%. (B) Weight was monitored during the course of study and represented as mean ± SD (*n* = 5–7). Leukemic burden in the (C) bone marrow, (D) spleen, and (E) peripheral blood 24 days postengraftment was measured using flow cytometry. REH cells were identified in single-cell tissue suspensions as human CD19+ and CD45+ (hCD19+CD45+) cells and compared to the percentage of all live cells counted. Data represented as individual points with a bar denoting the median of the group (*n* = 5–7). Significance was determined by Kruskal–Wallis ANOVA followed by posthoc Dunn’s multiple comparisons test, *p < 0.05. Panel A was created using BioRender (https://BioRender.com/42ni4dt).

Importantly, treatment with 0.02 and 0.05 mg/kg MMAE-LNPs did not result in significant reductions in weight during the treatment period, as seen previously with free 0.1 mg/kg MMAE (Figure 6B). Treatment of the primary leukemic site with MMAE-LNPs was subtherapeutic, with 0.0, 0.02, and 0.05 mg/kg groups exhibiting median bone marrow tumor burdens of 86, 82, and 71%, respectively (Figure 6C). While median tumor burden reduction in the bone marrow was not statistically significant following treatments, 2 out of the 7 mice treated with 0.05 mg/kg LNP-MMAE had bone marrow tumor burdens ≤ 1% demonstrating successful eradication in a subset of the treatment group. Further highlighting the potential therapeutic effect of MMAE-LNPs, the splenic and blood burdens for the 0.05 mg/kg LNP-MMAE treatment group were significantly lower than the 0.0 mg/kg control group. The median leukemic burdens were 2.0- and 6.1-fold lower within the spleen and blood, respectively, when comparing between the 0.0 and 0.05 mg/kg groups (Figure 6D,E).

Intravenous administration of leukemic cell lines within immunodeficient mice can initially engraft in regions outside of the bone, which deviates from direct clinical progression originating within the bone marrow. While further characterization for the progression of leukemia in xenograft models is needed, patient-derived xenografts have been shown to have consistent end point engraftment rates in clinically relevant sites for primary and relapsed disease, such as the bone marrow and spleen, based on their molecular profiles.^24,33–35,67,78,79^ Detection of leukemic cells within the blood is an invaluable quantification method to better elucidate the progression of disease in xenograft models and parallels what is done clinically for the monitoring of minimal residual disease.^80–82^

Nonetheless, even at subtherapeutic levels within the bone marrow for this study, MMAE-LNPs demonstrated a dose-dependent decrease in splenic burden and overall disease progression without exhibiting abrupt weight loss indicative of MMAE systemic toxicity. This therapeutic effect within the spleen may be attributed to the notable localization of MMAE-LNPs that was observed from our biodistribution studies. While not the primary site of disease for B-ALL, reduction of splenic burden remains clinically relevant for the treatment of extramedullary disease. In relation to the bone marrow, our biodistribution studies did not indicate enhanced accumulation of MMAE-LNPs as observed in the spleen. While the spleen and bone marrow contain fenestrated sinusoids, their physiologic purposes are unique between these organs. The liver and spleen are the primary organs comprising the MPS capable of engulfing and sequestering microorganisms, foreign particles, and aging circulatory cells.^83–85^ The bone marrow has also been demonstrated to have a minor but readily saturable role of particle sequestration, with its primary utilization of sinusoids being for ingress and egress of hematopoietic-derived cells.^86^ The size of bone marrow sinusoids is dynamic and tightly regulated, potentially limiting the extravasation of our 120 nm MMAE-LNPs within the bone marrow parenchyma.^87^ This further supports the need for research evaluating the addition of targeting moieties that are highly prevalent on the surface of B-ALL cells. Receptors such as CD19, CD22, or, more recently, the integrin very late antigen-4, all hold promise to further enhance nanoparticle specificity, accumulation, and retention within these sites.^27,88,89^ Furthermore, while our preliminary findings indicate no abrupt weight loss due to the MMAE-LNPs, future studies thoroughly evaluating the dose-dependent pharmacokinetics, histopathology of organs, and acute versus chronic exposures to MMAE-LNPs must be performed to further establish translational feasibility. Taken together, our proof-of-concept MMAE-LNP efficacy study established baseline antileukemic activity, which may be enhanced when tested as a combinatorial treatment utilizing functionally relevant siRNA therapeutics.

## 3 Conclusions

Although liposome formulations allow encapsulation of physicochemically diverse small-molecule therapeutics, their vesicular morphology limits the dual encapsulation of nucleic acids and small-molecule therapeutics. While ionizable LNPs have been meticulously engineered to effectively entrap nucleic acid therapeutics, exploiting the surplus of lipid-rich domains in the solid-dense internal structure of LNPs for small molecule loading remains largely unexplored. In this work, we successfully coloaded MMAE and inert siRNA within clinically relevant ionizable LNPs. We have established an optimized method to effectively encapsulate MMAE within prefabricated LNPs containing negative-control siRNA, which displayed unaltered physicochemical characteristics, stability, and siRNA retention. Ionizable LNPs were efficiently taken up by B-ALL cells *in vitro* with uptake inhibitor studies indicating the involvement of macropinocytosis and clathrin-mediated endocytosis. MMAE-LNPs preserved the potency of MMAE and demonstrated superior antileukemic activity compared to the standard-of-care agent, vincristine, in B-ALL cells. MMAE encapsulation did not alter the inherent biodistribution of LNPs, with the liver and spleen as the two most prevalent locations for LNP accumulation. In a B-ALL human xenograft mouse model, MMAE-LNPs demonstrated a significant dose-dependent decrease in the leukemic burden within the spleen and blood, without showing overt systemic toxicity exhibited by free MMAE. Given that the dosing regimen employed in this study did not result in a significant reduction of the bone marrow leukemic burden, our findings provide a critical basis for future studies that focus on enhancing accumulation and retention of LNPs in the bone marrow. Furthermore, given the universal role tubulin polymerization has in cell division, nonspecific inhibition by antimitotic agents may lead to undesirable off-target effects. The significant and indiscriminate cellular potency of MMAE necessitates studies evaluating targeted delivery to leukemia cells to clinically enhance the therapeutic window for this chemotherapy loaded within LNPs.^90^ Of note, the full potential of this platform for B-ALL treatment could be unveiled by investigating functional siRNA therapeutics against genetic targets essential to leukemic viability. Future experimentation will require quantitative techniques, such as western blotting or flow cytometry, to thoroughly evaluate the protein-level knockdown achievable with this platform and observe the potential synergies with loaded MMAE. Together, our findings provide critical insight into developing versatile platforms to simultaneously codeliver disparate treatment modalities that can enhance treatment efficacy for aggressive malignancies.

## 4 Experimental Section

### 4.1 Fabrication of LNPs

LNPs used throughout all experiments were created with a flash nanoprecipitation (FNP) technique utilizing a confined impingement jet (CIJ) mixer as previously described.^31^ The composition of LNPs was calculated for total lipid amounts for 20 mM formulations with molar ratios of 50:38.5:10:1.5 for DLin-MC3-DMA (MedChemExpress), cholesterol (Alfa Aesar), DSPC (Cayman Chemical), and DMG-PEG(2000) (Cayman Chemical), respectively. Lipid components were dissolved in absolute ethanol, while the negative control siRNA (NC-siRNA, MedChemExpress) utilized was dissolved in RNAase-free 50 mM sodium citrate buffer (pH = 4.0). To ensure sufficient packing of siRNA within LNPs, the nitrogen-phosphate (N:P) ratio was between 6 to 10 for all formulations. For the tracking of siRNA payloads, 15% of the standard negative control siRNA was replaced with Mission Cy5-conjugated negative control siRNA (Sigma-Aldrich). Hydrophobic membrane tracers such as 1,1′-dioctadecyl-3,3,3′,3′-tetramethylindodicarbocyanine, 4-chlorobenzenesulfonate salt (DiD) and indocyanine green (ICG) (MedChemExpress), 0.075 and 0.5 mg were loaded in the organic phase prior to FNP impingement, respectively.

The organic and aqueous phases were manually impinged through a CIJ mixer and quenched with sodium citrate buffer. Overnight dialysis with formulations in SnakeSkin dialysis membrane (10,000 MWCO, Thermo Fischer Scientific) was done to remove ethanol and perform a buffer exchange. LNPs not designated for Monomethyl Auristatin E (MMAE, MedChemExpress) postfabrication loading were dialyzed in sterile phosphate-buffered saline (PBS) (pH = 7.4, Gibco) while formulations utilized for MMAE loading were dialyzed in 0.1 M carbonate-bicarbonate buffer (pH = 10.8). A Sephadex LH-20 filtration column (Cytiva) equilibrated to the same buffer was used to remove unbound tracer after dialysis.

LNPs that underwent MMAE postfabrication loading were initially concentrated to a total lipid concentration ranging from 10 to 20 mg/mL using a 100 kDa Amicon Ultra Centrifugal Filter (Sigma-Aldrich). MMAE dissolved in methanol, Optima LC/MS grade (Thermo Fischer Scientific), was incorporated with LNPs at a drug-to-lipid ratio of 0.015–0.06 (<5% total volume) and allowed to incubate for 2h on a MultiTherm shaker (Benchmark Scientific) set to 500 rpm at 4 °C. Unloaded MMAE was removed by washing the LNPs with 8 volumes of sterile PBS through a 100 kDa Amicon Ultra centrifugal filter. All formulations for *in vivo* experimentation were filtered using a sterile Poly­(ether sulfone) 0.2 μm filter (Thermo Fisher Scientific).

### 4.2 Characterization of LNPs

Hydrodynamic size, polydispersity index (PDI), and zeta potential for LNP samples were determined at a dilution of 1:50 in PBS using a Zetasizer Ultra (Malvern Panalytical). Transmission electron microscopy images were taken on carbon-coated EM grids (Ted Pella, 01840-F) and captured using a JEOL 1010 microscope with an AMT XR611S–B CCD camera as previously described.^91^ Likewise, a Glacios cryo-transmission electron microscope (Thermo Fisher Scientific) with C-Flat 1.2/1.3 standard 20 nm carbon film adhered to 300 copper mesh grids was used as previously described to acquire representative micrographs.^92^

The effect of MMAE loading on the apparent p*K*_a_ of the LNP was evaluated using a 6-p-Toluidino-2-naphthalenesufonic acid (TNS) fluorescent-based assay as previously described.^31,92^ In brief, the polyprotic buffer used for the assay was created with deionized water and 20 mM sodium monophosphate, 20 mM ammonium acetate, and 25 mM citrate, and 150 mM sodium chloride. Incremental aliquots of the buffer at varying pHs were created using sodium hydroxide, ranging from 3.5 to 9.5, and validated using an accumet AE150 Benchtop pH meter (Thermo Fisher Scientific). At each pH, the fluorescence of 6 μM TNS was evaluated with or without 20 μM of total lipid concentration from the LNP. Fluorescence was measured at an excitation–emission of 322/431 nm using a SpectraMax iD5 (Molecular Devices) microplate reader. The apparent p*K*_a_, i.e the half-maximal fluorescent intensity, was extrapolated using a sigmoidal nonlinear regression in GraphPad Prism (version 10.2.3).

### 4.3 Quantification of siRNA and MMAE

Encapsulation of RNA was determined using the QuantiFluor RNA System in tandem with the Quantus Fluorometer (Promega). Samples were diluted 1:10 in RNase-free water containing 1xTE buffer (pH 7.5) with or without 1x Triton (Thermo Fischer Scientific). Samples were incubated at 37 °C in an Incu-Shaker Mini (Benchmark Scientific) set to 150 rpm for 10 min. Quantification of siRNA was determined following Promega’s manufacturer instructions. The relative encapsulation efficiency, representing the percentage of complexed siRNA within the formulated sample, was determined by the following eq 1

1 $$\mathrm{EE}=\frac{{\left[\mathrm{siRNA}\right]}_{+\mathrm{Triton}}-{\left[\mathrm{siRNA}\right]}_{-\mathrm{Triton}}}{{\left[\mathrm{siRNA}\right]}_{+\mathrm{Triton}}}$$

where the numerator represents the concentration of encapsulated siRNA. All samples were quantified in experimental duplicate.

For the quantification of MMAE released or within filtered LNPs, samples diluted within sterilized distilled water (Gibco) followed by flash freezing at −80 °C for 45 min. Lyophilization of samples was done using a Lyovapor L-200 (BUCHI) set at −50 °C and 0.2 mbar for 24h. Lyophilized samples were resuspended using methanol and vortexing, with precipitates removed through centrifugation at 10,000 g x 5 min. Samples were stored at −20 °C until quantified using liquid chromatography-tandem mass spectrometry (LC-MS/MS).

LC was done using an ExionLC Series Ultra High-Pressure Liquid Chromatograph (AB Sciex). The mobile phase was composed of 80.0% acetonitrile and 20.0% water, both containing 0.1% formic acid. The flow injection volume was 2.0 μL with separation done at an isocratic flow rate of 0.400 mL/min through a Phenomenex column (Torrance, CA, USA) LunaOmega (1.6 μm C18 100 Å) LC Column (100 × 2.1 mm). The oven was maintained at 40 °C and the cooler temperature was 7 °C. The control vial needle had a stroke depth of 48 mm. MS was conducted using a Qtrap 5500 Mass Spectrometer (AB Sciex). MMAE was detected using positive electron spray ionization (ESI+) with multiple reaction monitoring peaks integrated using MultiQuant software (AB Sciex). Quantification was conducted by monitoring the transition from precursor ions of MMAE (*m*/*z* 719.1) to product ions (*m*/*z* 126.9). A dwell time of 50.0 ms was used to monitor mass transitions, with N_2_ as the collision gas. The declustering potential, entrance potential, collision energy, and collision exit potential were set to 90.0 v, 10 v, 41 v, and 13 v, respectively, in all trials. The total run time was 4 min per sample with a quantifiable range for MMAE samples of 20–1400 ng/mL.

### 4.4 Storage and Stability of LNPs

To evaluate the optimal storage conditions of fabricated LNPs loaded with MMAE, formulations were stored in varying conditions. Physicochemical characterization of LNPs loaded with MMAE, was done shortly after creation. Standard shelf life storage at 4 °C was evaluated after 1-month. Removal of unbound or released MMAE in stored samples was accomplished as previously described. Physicochemical characterization of stored samples was then re-evaluated to determine the change in particle size, and the release of loaded NC-siRNA and MMAE was quantified using the methods described above. siRNA retention was importantly determined by comparing the encapsulated siRNA concentrations at initial and final time points. MMAE retention in LNPs was determined by quantifying the total amount of MMAE present after 1 month before and after removal of unbound MMAE. All LNPs utilized for *in vitro* and *in vivo* experimentations were stored at 4 °C and used within 2 weeks of fabrication.

To determine the release of MMAE loaded in LNPs when placed in serum-like conditions, MMAE-LNPs were diluted 1:10 in PBS at a final concentration of 10% fetal bovine serum (FBS; Corning). Samples were allowed to incubate at 37 °C up to 48 h with constant shaking (150 rpm) in a MultiTherm shaker. Samples were rinsed through an 100 kDa Amicon Ultra Centrifugal Filter with 4 mL of PBS and the supernatant was checked for release of MMAE following the LC-MS/MS quantification methods described above.

### 4.5 Cell Culture

Human B-ALL suspension cell lines REH and SUPB15 were acquired through the American Type Culture Collection (ATCC, CRL-8286 and CRL-1929). REH cells were suspended in fresh Roswell Park Memorial Institute (RPMI) 1640 (Gibco) complete growth medium, comprised of 89% base medium, 10% FBS, and 1% Penicillin-Streptomycin (PenStrep) (Gibco). SUPB15 cells were suspended in fresh Iscove’s Modified Dulbecco’s Medium (IMDM) (Cytiva) with 4 mM l-glutamine adjusted to contain 1.5 g/L sodium bicarbonate and supplemented with 0.05 mM 2-mercaptoethanol (Gibco), comprised of 79% base medium, 20% FBS, and 1% PenStrep. Cell lines were maintained according to the handling information section on the ATCC Web site and incubated at 37 °C with 5% CO_2_. MycoStrip (Invivogen) test strips were used to check the cell lines for mycoplasma contamination every 2–3 months following the manufacturer’s protocol.

### 4.6 Cell Viability Studies

For all viability studies performed, REH and SUPB15 cell lines were plated in black, clear-bottom 96-well plates (Thermo Fisher) with 5000 cells per well in 100 μL of respective cell media. Inherent toxicity of LNPs was determined after 72h incubation at varying concentrations of negative-control siRNA loaded within the formulations. For toxicity studies utilizing chemotherapies, cell viabilities were determined after 48h incubations with serial dilutions of MMAE (<1% DMSO in well), vincristine, or MMAE-loaded LNPs. Toxicity of all compounds or formulations was measured using the luminescent-based CellTiter-Glo 2.0 Cell Viability Assay (Promega) using a SpectraMax iD5 microplate reader following the manufacturer’s assay instructions. Viabilities were calculated by normalizing treated wells with PBS-treated cells.

### 4.7 *In Vitro* LNP Uptake and Transfection Capability

For inherent uptake studies, REH or SUPB15 cells were plated in 96-well U-bottom plates (VWR) at a concentration of 1 × 10^5^ cells/mL and incubated with DiD-loaded LNPs following a 30 min incubation with PBS or endocytic inhibitors, chlorpromazine (CPZ) and amiloride (AMIL) (Cayman Chemical), at time points of 1h, 4h, and 24h. CPZ was plated at concentrations of 25 μM and 50 μM per well, and AMIL was plated at a concentration of 50 μM per well. LNPs were plated at an siRNA concentration of 50 nM per well. Transfection studies were performed similarly, with cells plated in 96-well U-bottom plates at a concentration of 1 × 10^5^ cells/mL and incubated with LNPs containing Cy5-conjugated siRNA (50 nM in well) at time points of 1, 4, and 24h. Following the incubation periods, cells were prepared for flow cytometry and confocal imaging to assess the uptake and localization of the LNPs.

Prior to flow cytometry, cells were washed twice with PBS, resuspended in Cell Staining Buffer (Biolegend), stained with zombie red fixable viability dye (Biolegend), and fixed using Fixation Buffer (Biolegend). A 3-Laser Cytek Aurora flow cytometer controlled with SpectroFlo software (Cytek Biosciences) was used for data acquisition and with over 30,000 ungated events collected per sample. The live cell populations were analyzed using Cytobank (version 10.7) with relative median fluorescent intensity (relative MFI) representing the MFIs for samples normalized to the brightest incubation group, and Cy5+/DiD+ population gating was determined using single stains and PBS controls.

To prepare for confocal imaging, samples were washed twice with PBS, fixed using Fixation Buffer (Biolegend), and resuspended in PBS at a concentration of 3 × 10^5^ cells/mL. The fixed cells were transferred to Colorfrost Plus Adhesion Microscope slides using Cytofunnels (Epredia) loaded into a Cytospin 2 slide centrifuge (Thermo Fisher Scientific) set at 750 rpm for 8 min. ProLong Diamond Antifade Mountant with DAPI (Invitrogen) was applied to each slide and along with a coverslip, and slides settled overnight at room temperature away from light prior to imaging. Localization of fluorescent LNPs or siRNA was imaged using a Zeiss LSM 710 Confocal Microscope with 405 nm (DAPI) and 640 nm (DiD and Cy5) lasers along with bright field. Images within the same figure panels were taken using the same laser powers and detector settings. Images were processed with ImageJ software (Version 2.14.0) such that the intensities of the Cy5/DiD channel are directly comparable between respective image groups.

Imaging flow cytometry with REH cells was performed with Amnis ImageStream cytometer (Cytek Biosciences). In addition to necessary single-stain controls, 25 nM of Cy5-conjugated siRNA in LNPs was incubated for 24 h in 24-well nontreated plates containing 1 million REH cells. Cells were washed with PBS and nonspecific binding was blocked using human serum IgG (Sigma-Aldrich). Treatment groups were stained with mouse PE-conjugated anti-CD19 antibodies (HIB19 clone; Thermo Fisher Scientific) and LysoView 488 lysosomal stain (Biotium) following manufacturers’ protocols. Cells were then fixed and stained with NucBlue Fixed Cell ReadyProbes DAPI Reagent (Thermo Fisher Scientific) prior to imaging. Instrumentation for imaging flow cytometry was controlled using the INSPIRE software with collection set to 10,000 events for single, focused cells positive for CD19 and DAPI. Data analysis was performed with the IDEAS software, evaluating single, focused cells positive for CD19, DAPI, Lysosome, and Cy5-siRNA. Subcellular localization of Cy5-siRNA was determined with creating a series of masks. In brief, a cytoplasm mask was created with Boolean subtraction between a whole-cell mask (brightfield) and nuclear mask (DAPI). A lysosomal spot mask isolating lysosomal fluorescence was then applied to the cytoplasm mask. The percentage of Cy5 intensity within the cytoplasm and lysosome was quantified and representative images of the analyzed population were acquired.

### 4.8 Animal Experimentation

All *in vivo* studies conducted were approved by the West Virginia University Institution of Animal Care and Use Committee (Protocol #2306066797). For biodistribution studies, mice were monitored daily for discomfort and weight loss after injection. For xenograft leukemia model studies, clinical end points included altered thirst, anemia, absence of ambulation, hind-limb paralysis, and weight loss greater than 10% of the original weight. Mice meeting any of these predetermined end points were promptly and ethically euthanized following the institution’s guidelines.

### 4.9 Biodistribution of LNPs with and without MMAE

6-week-old male BALB/c mice (The Jackson Laboratory) were intravenously injected with 100 μL PBS or LNPs with or without MMAE (0.3 mg/kg). Both formulations were also loaded with ICG and NC-siRNA (2.0 mg/kg). At end points of 4 or 24 h, serum was collected via intracardiac puncture followed by euthanasia under isoflurane (Pivetal) and organs were perfused with 15 mL PBS. The femur, tibia, liver, spleen, kidneys, lungs, heart, and brain were then collected. The total radiant efficiency ((p/s)/(μW/cm2)) of ICG loaded in LNPs was measured using the IVIS SpectrumCT (PerkinElmer) controlled with Living Image software (Caliper LifeSciences) set at medium binning with an exposure time ranging from 7 to 30 s.

The relative total radiant efficiency was determined for each organ by dividing the measurements by the averaged total radiant efficiency of the PBS group for that respective organ. Likewise, when calculating the percentage of signal for each organ, the averaged PBS signal representing that organ was first subtracted. For each mouse, the background-corrected organ signals were summed. The organ distribution percentage for each mouse organ was then calculated as the proportion of the individual organ signal to mouse’s summed signal. Calculated values for organ distribution percentages were averaged between their groups.^93^

### 4.10 REH Tumor Burden Efficacy Study

6-week-old male NSG mice (WVU Transgenic Animal Core Facility) were engrafted intravenously with 2 million REH cells with mice being randomized into treatment groups by body weight. At 7 days postengraftment, mice given treatments consisting of four intravenous injections administered once every 3 days. Treatment groups included MMAE-loaded LNPs at 0.02 and 0.05 mg/kg as well as a control group injected with LNPs (0.0 mg/kg MMAE). Notably all LNP formulations in this study contained negative-control siRNA (0.3 mg/kg). Four weeks postengraftment, mice were euthanized with blood, spleen, femurs, and tibias collected. For preliminary MMAE dosing studies, free MMAE (0.1 mg/kg) or PBS were administered following the same dosing regimen. For all the dosing regimens containing MMAE, mice were carefully monitored for weight loss throughout the study with euthanasia performed based on the ethical guidelines described previously. At the end of the efficacy study, tissues were processed to create a single cell suspension for flow cytometry using red blood cell lysis buffer (BioLegend) following the manufacturer’s protocol. Nonspecific binding was blocked using antimouse CD16/32 antibody (BioLegend) and human serum IgG (Sigma-Aldrich). Flow staining included zombie red (BioLegend) and mouse PE-CF594 conjugated anti-CD19 (HIB19 clone) and mouse APC-R700 conjugated anti-CD45 (HI30 clone) from BD Biosciences. Samples were run through a 3-Laser Cytek Aurora flow cytometer and the ungated events collected were 5,000–50,000 for lysed blood, 200,000 cells for spleen, 100,000 cells for bone marrow. Flow data was analyzed using Cytobank (Version 10.7). REH burden was determined by evaluating the percentage of hCD19+CD45+ cells within the live cell population for the samples.

### 4.11 Statistical Analysis

The software utilized to perform statistical analyses throughout this manuscript was GraphPad Prism (version 10.2.3). All studies were performed in experimental replicates of at least 3 with data presented as mean ± SD, unless specified otherwise, and significance throughout all studies was set to a p-value <0.05. Statistical analyses included ordinary unpaired *t* tests, unpaired Mann–Whitney rank test, one-way ANOVA followed by posthoc Tukey’s multiple comparisons test, and Kruskal–Wallis one-way ANOVA with Dunn’s multiple comparisons test.

## Supplementary Material



## Notes

Additional MMAE postfabrication loading characterization; siRNA retention and transmission electron micrographs; stability and release kinetics of MMAE-LNPs; inherent toxicity of siRNA-LNPs in B-ALL cells; subcellular localization of siRNA-LNPs in REH cells; additional confocal images for LNP and siRNA localization in B-ALL cells; percentage of organ radiant efficiency between siRNA-LNPs ± MMAE with statistical analysis; and preliminary efficacy and tolerability of unbound MMAE in human xenograft model (PDF)
