# Supplementary material for: Delivery of Monomethyl Auristatin E Using Ionizable Lipid Nanoparticles for B‑Cell Acute Lymphoblastic Leukemia Treatment
Source: ACS Omega. 2026 May 25;11(22):32321–35. doi: 10.1021/acsomega.5c13457 (PMC13261410; doi:10.1021/acsomega.5c13457)
Supplement: Supplementary file 1 [file ao5c13457_si_001.pdf]

Supporting Information

Delivery of Monomethyl Auristatin E Using Ionizable Lipid Nanoparticles for B-cell Acute Lymphoblastic Leukemia Treatment

*William H. Pentz[1,2], Krystal A. Hughes[1], Bishal Misra[1], Srikiran V. Nandigama[3] , Aidan Murray[4], Aimery Samuelson[5], Morgan Surface[6], Rukiye Nur Akpolat-Seker[1], Boopalan Sivanathan[1], and *Sharan Bobbala[1,7]*

[1]*Department of Pharmaceutical Sciences, West Virginia University School of Pharmacy, Morgantown, WV 26506, USA*

[2]*School of Medicine, West Virginia University, Morgantown, West Virginia, 26506, USA*

[3]*Department of Chemistry and Biochemistry, West Virginia Wesleyan College, Buckhannon, WV 26201, USA*

[4]*Department of Microbiology, Immunology and Cell Biology, West Virginia University School of Medicine, Morgantown, WV 26506, USA*

[5]*Department of Chemistry, Berea College, Berea, KY 40404, USA.*

[6]*Department of Clinical Pharmacy, West Virginia University School of Pharmacy, Morgantown, WV 26506, USA*

[7]*West Virginia University Cancer Institute, Morgantown, WV 26506, USA*

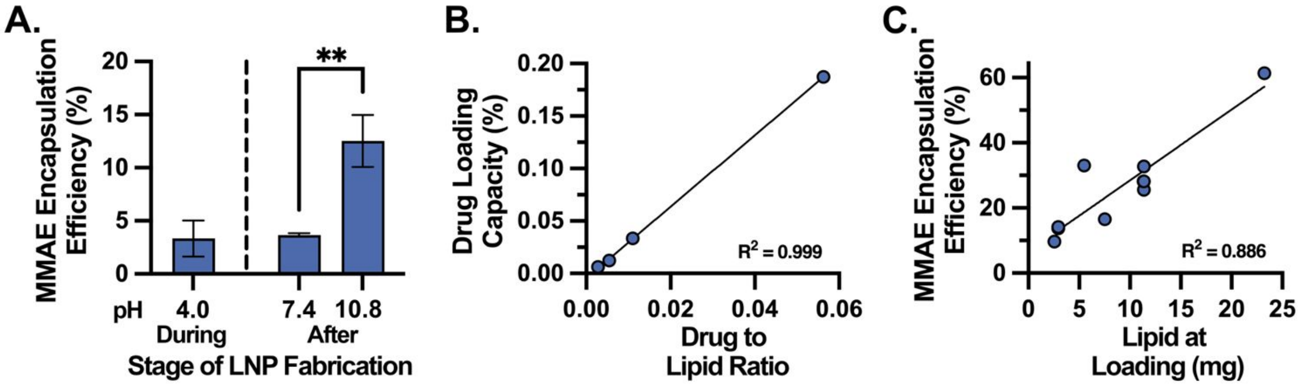

Figure S1. Optimization and feasible scaling of MMAE post-fabrication loading. (**A**) Attempt to load MMAE during (left) and after (right) LNP fabrication with flash nanoprecipitation. The notated pH is indicative at the time of MMAE loading. Data represented as mean ± SD (n=3). Significance determined with unpaired t-test, ** $p < 0.01$. (**B**) Proportional increase in the drug loading capacity of MMAE within siRNA-LNPs from drug to lipid ratios of 0.003 to 0.06. (**C**) The presence of more total lipid mass during remote loading after pH 10.8 dialysis demonstrates a positive linear trend regardless of drug-to-lipid ratio. Linear regressions were performed with the least-squares method.

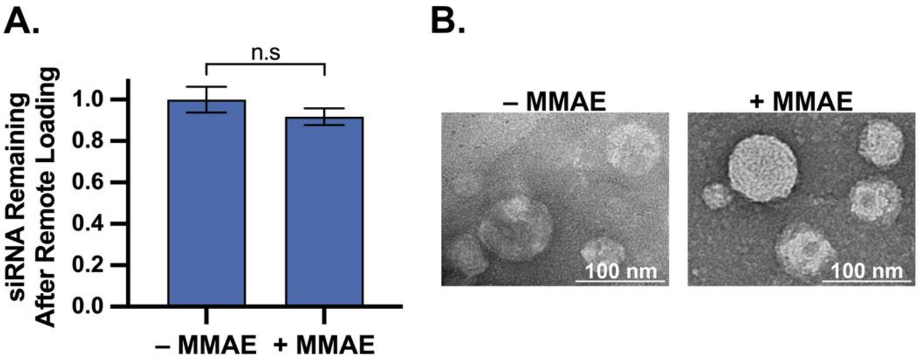

Figure S2. Additional characterization of siRNA-LNPs remote loaded with MMAE. (**A**) Proportion of siRNA retained after remote loading process with MMAE. Data represented as mean ± SD (n=3). Significance was evaluated with ordinary unpaired t-tests ($p > 0.05$). (**B**) Negative-stain transmission electron microscopy of LNPs without and with MMAE remote loading. The scale bar is 100 nm.

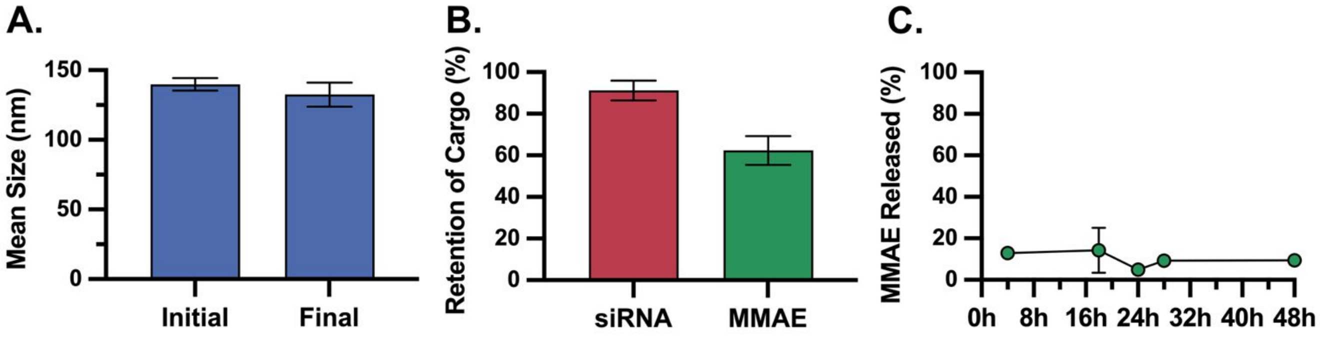

Figure S3. Month-stability and release kinetics of MMAE-LNPs. (**A**) Mean size of MMAE-LNPs immediately after fabrication (initial) and 1-month after 4ºC storage (final). (**B**) Retention of siRNA (red) and MMAE (green) after 1-month 4ºC storage with MMAE-LNPs was measured. (**C**) Kinetic release profile of MMAE from MMAE-LNPs in PBS containing 10% FBS at 37ºC with constant shaking at various timepoints. Mean size was measured using dynamic light scattering, while amounts of siRNA and MMAE were quantified using Quantifluor RNA system-based assay and LC-MS/MS, respectively. All data presented as mean ± SD (n=3). Significance for MMAE release between timepoints was evaluated with one-way ANOVA and post-hoc Tukey's multiple comparison ($p > 0.05$).

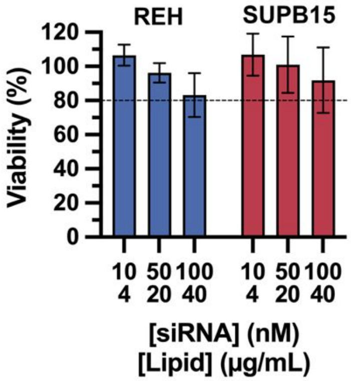

Figure S4. Inherent in vitro toxicity of LNPs loaded with negative control siRNA in REH (blue) and SUPB15 (red) cells after 72-hour incubation. Total siRNA (top) and lipid concentration (bottom) in the well are indicated on the x-axis. Viability was determined using CellTiter-Glo 2.0. Data represented as mean ± SD (n=4).

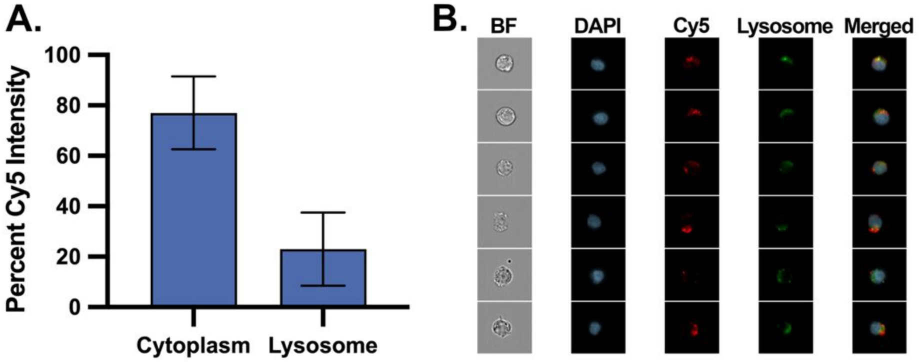

Figure S5. Subcellular localization of Cy5-conjugated siRNA in REH cells. REH cells were treated with Cy5-siRNA LNPs for 24 hours. (**A**) Imaging flow cytometry was used to quantify the percent of Cy5 intensity subcellularly located within the cytoplasm or the lysosomes. (**B**) Representative 33 x 33 µm images of REH cell population analyzed with brightfield (BF), nuclei (DAPI), Cy5-siRNA (Cy5), lysosome, and merged fluorescent images. Data represented as mean ± SD (n=9,754).

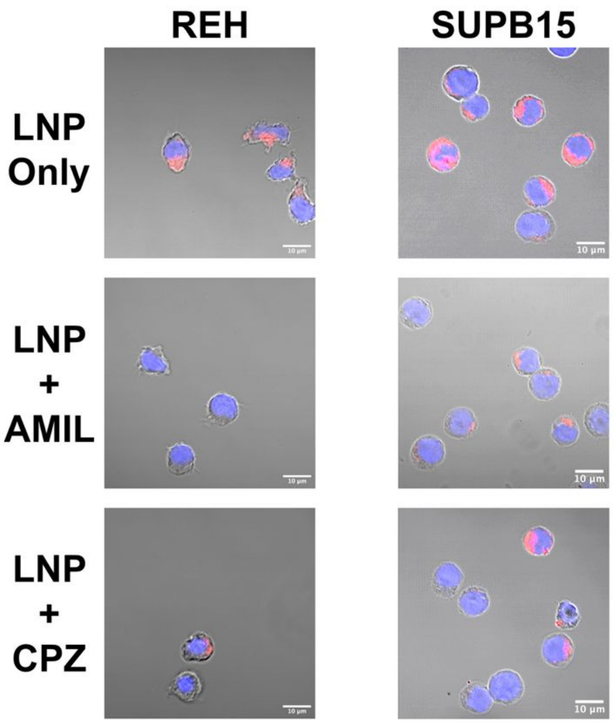

Figure S6. Additional confocal images for localization of DiD-loaded LNPs in REH and SUPB15 cells. DiD-loaded LNPs with negative control siRNA were incubated in wells containing REH or SUPB15 cells for 24 hours following 30-minute incubation with PBS, amiloride (AMIL), or chlorpromazine (CPZ). Merged images provided with brightfield, DAPI (blue), and DiD (red). Scale bars represent 10 μm.

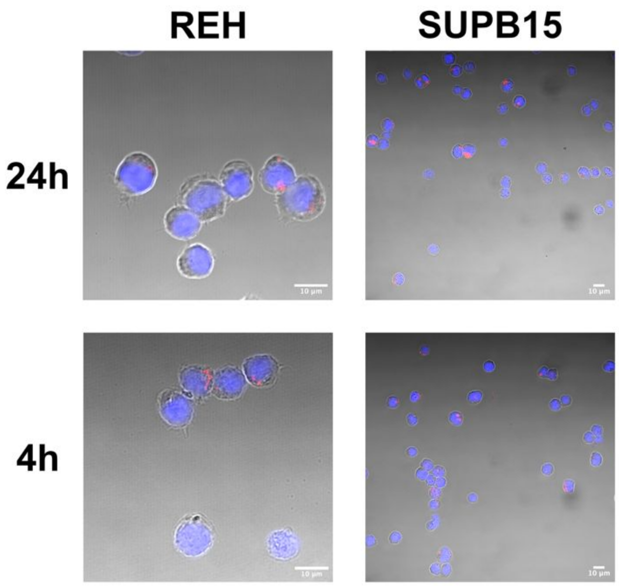

Figure S7. Additional confocal images for localization of Cy5-siRNA LNPs in REH and SUPB15 cells. LNPs loaded with Cy5-siRNA were incubated in wells containing REH or SUPB15 cells for 24 or 4 hours. Merged images provided with brightfield, DAPI (blue), and DiD (red). Scale bars represent 10 µm.

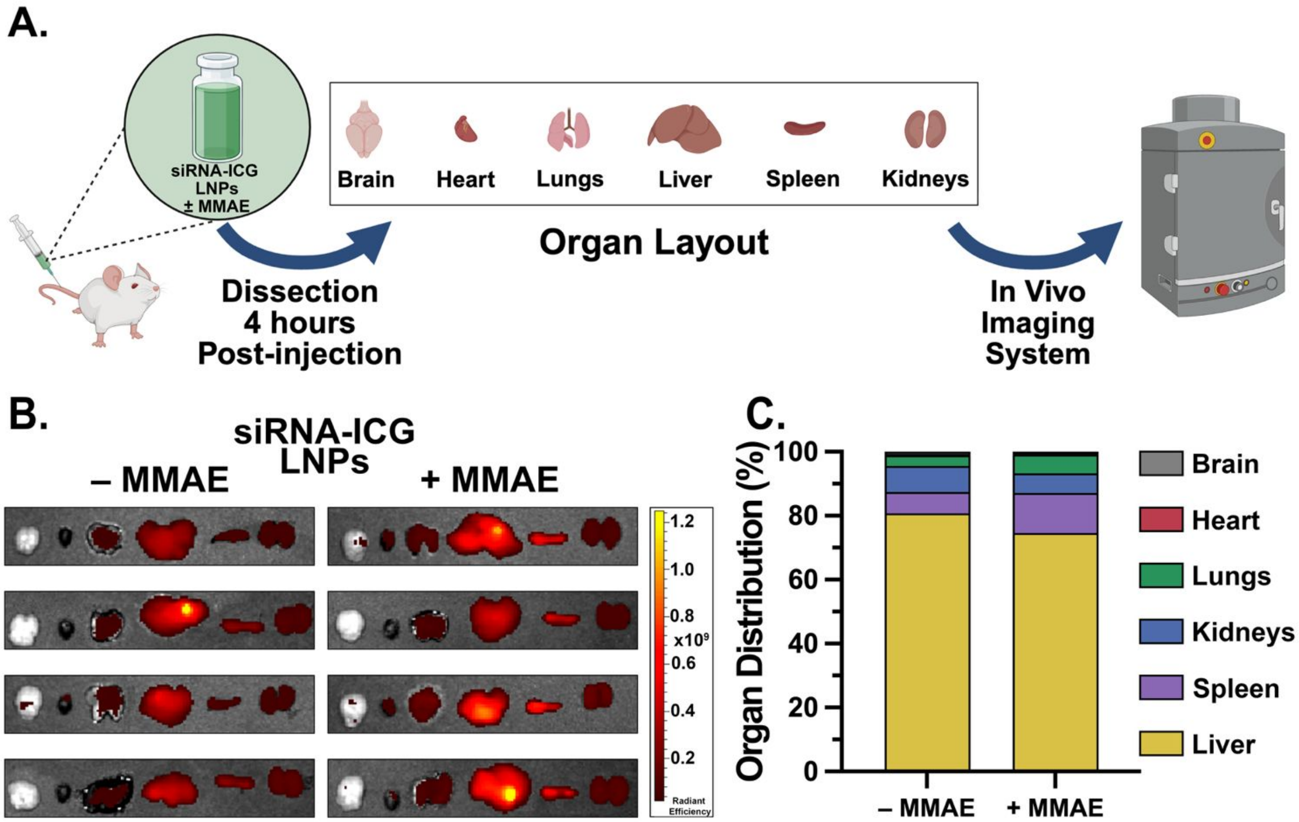

Figure S8. Organ biodistribution of LNPs with and without MMAE. Mice were intravenously injected with ICG-labeled LNPs and dosing was matched with loaded siRNA (2.0 mg/kg) in both formulations. MMAE-loaded LNP also contained MMAE (0.3 mg/kg). (**A**) *Ex vivo* analysis was performed at 4 hours. (**B**) Visualization of radiant efficiency ([p/sec/cm^2/sr]/[μW/cm^2]) for the brain, heart, lungs, liver, spleen, and kidneys. (**C**) The organ distribution (%) represents the proportion of radiant efficiency for each mouse organ after subtracting the average background fluorescence from each relevant organ in the PBS group (n=3). Data represented as mean with the legend ranking lowest to highest organ accumulation from top to bottom, respectively (n=4). Statistical analysis for each organ distribution (%) can be found in **Supplemental 9**. Panel A was created using BioRender (https://BioRender.com/2w6w67c).

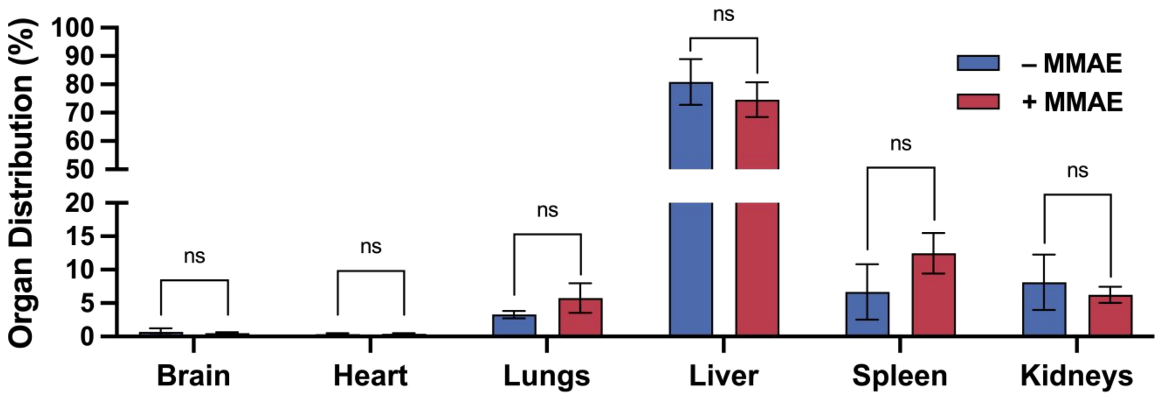

Figure S9. Percentage of organ radiant efficiency (Organ Distribution (%)) in mice injected with ICG-labeled LNPs with or without MMAE. Values for each mouse were calculated after subtracting the background radiant efficiency ([p/sec/cm^2/sr]/[μW/cm^2]) from each respective organ in the PBS group (n=3). Data represented as mean ± SD (n=4). Significance between groups was determined by ordinary unpaired t-tests (p > 0.05).

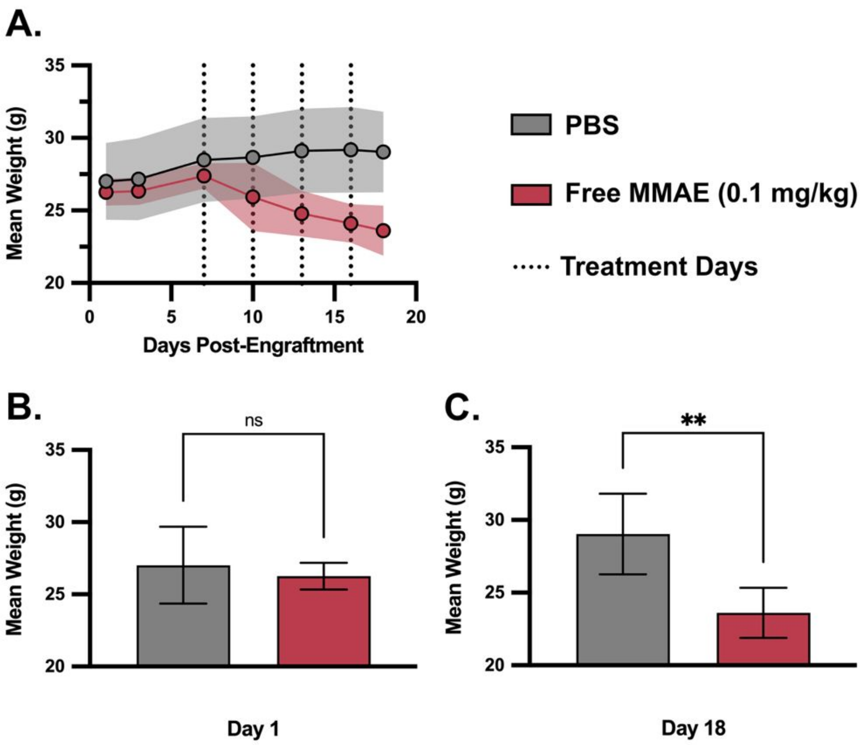

Figure S10. Preliminary tolerability of MMAE in a human xenograft model. NSG mice were engrafted with 2 million REH cells. Treatments of intravenous PBS (n=5) or 0.1 mg/kg free MMAE (n=6) began 7-days after engraftment and continued every 3 days for a total of 4 doses. (**A**) Weights were monitored throughout the study. (**B-C**) Mice treated with MMAE exhibited rapid weight loss, with a significant reduction in mean weight compared to control mice, and were ethically euthanized once a weight loss from baseline of 10% was achieved. Significance was determined by an ordinary unpaired t-test, $^{**}p < 0.01$. All data are represented as mean ± SD.
